# A Population-Based Study of Social Demographic Factors, Associated Diseases and Recurrent Corneal Erosion in Taiwan

**DOI:** 10.3389/fpubh.2022.832333

**Published:** 2022-03-28

**Authors:** Ren-Long Jan, Chung-Han Ho, Jhi-Joung Wang, Sung-Huei Tseng, Yuh-Shin Chang

**Affiliations:** ^1^College of Health Sciences, Graduate Institute of Medical Sciences, Chang Jung Christian University, Tainan, Taiwan; ^2^Department of Pediatrics, Chi Mei Medical Center, Liouying, Tainan, Taiwan; ^3^Department of Hospital and Health Care Administration, Chia Nan University of Pharmacy and Science, Tainan, Taiwan; ^4^Department of Medical Research, Chi Mei Medical Center, Tainan, Taiwan; ^5^Department of Anesthesiology, Chi Mei Medical Center, Tainan, Taiwan; ^6^AI Biomed Center, Southern Taiwan University of Science and Technology, Tainan, Taiwan; ^7^Department of Ophthalmology, College of Medicine, National Cheng Kung University Hospital, National Cheng Kung University, Tainan, Taiwan; ^8^Department of Ophthalmology, Chi Mei Medical Center, Tainan, Taiwan

**Keywords:** recurrent corneal erosion, ocular allergic conditions, case-controlled study, Taiwan Longitudinal Health Insurance Database, diabetes mellitus, atopy

## Abstract

**Purpose:**

To investigate the association of recurrent corneal erosion (RCE) with sociodemographic factors and associated ocular conditions or systemic diseases.

**Methods:**

This nationwide, population-based, retrospective, matched case-controlled study included 98,895 RCE patients, identified by the International Classification of Diseases, Ninth Revision, Clinical Modification (ICD-9-CM) code 371.42, were selected from the Taiwan National Health Insurance Research Database. The age-, sex-, and index date- matched control group included 98,895 non-RCE control group also selected from the Taiwan Longitudinal Health Insurance Database 2000. Sociodemographic factors and associated ocular conditions or systemic diseases were examined using univariate logistic regression analyses, and continuous variables were analyzed using paired *t*-test. The odds ratio (OR) of developing RCE were compared using adjusted logistic regression analysis.

**Results:**

Patients with ocular conditions including corneal abrasion, ocular allergic conditions, and corneal dystrophy were more likely to have RCE than the control group (adjusted OR = 63.56, 95% CI = 42.06–96.06, *p* < 0.0001; adjusted OR = 24.27, 95% CI = 20.51–28.72, *p* < 0.0001; adjusted OR = 17.10, 95% CI = 5.14–59.93, *p* < 0.0001, respectively). Patients with systemic diseases such as diabetes mellitus, hyperlipidaemia, and atopy trait have significantly higher ORs for RCE development. Patients residing in either Northern Taiwan or a metropolis city had higher odds of developing RCE; however, there were no significant differences in income or occupation on the probability to develop RCE.

**Conclusion:**

RCE is strongly associated with corneal abrasion, ocular allergic conditions, corneal dystrophy, diabetes mellitus, hyperlipidaemia, and atopy trait.

## Introduction

Recurrent corneal erosion (RCE) is characterized by recurrent detachment of the corneal epithelium from the basement membrane of the eye. The most important risk factors for RCE include mechanical or surgical trauma to the corneal epithelium and corneal epithelial basement membrane dystrophies (1, 2). RCE typically occurs in eyes that have suffered a sudden, sharp, abrading injury by fingernail, paper cut, or tree branch (1, 3). Clinical presentations of RCE including sudden onset of eye pain, accompanied redness, photophobia, and tearing. These episodes vary in duration and severity.

Although poor adhesion of the corneal epithelium in corneal dystrophy has been a reported mechanism of RCE (4), the pathophysiology has yet to be fully determined. In addition, inflammation related to corneal surface injury or rubbing eyes weakens the extra-cellular adhesion network, disrupts the basement membrane, and activates gelatinase which may play a role in the RCE development (5). Recently, the imbalance between growth factors and adhesion molecules (6), as well as poor corneal sensitivity and tear function are reasons for an impaired wound healing response in RCE (7).

A key challenge to understanding RCE is the paucity of population-based epidemiologic study, and limited studies that focus on the associations of ocular or systemic conditions with RCE. Ocular trauma and diabetes mellitus (DM) are important causes of RCE (1). Notably, atopic keratoconjunctivitis and DM are significant risk factors of RCE (8).

The purpose of this study was to use a health care claims database containing records for more than 90,000 RCE patients and control group matched by age, sex, and index date to investigate the association between RCE and sociodemographic factors, various ocular or systemic comorbid conditions, and to elucidate its pathophysiologic features.

## Materials and Methods

### Database

Medical records were taken from the National Health Insurance Research Database (NHIRD), provided by the National Health Research Institute (NHRI) of Taiwan. The NHIRD provided encrypted patient identification numbers together with information on patient demographics such as date of birth, sex, place of residence, and dates of admission or discharge. It also incorporated the International Classification of Diseases, Ninth Revision, Clinical Modification (ICD-9-CM) codes, which recorded prescription items, diagnoses and procedures, as well as costs covered by the NHRI. The research was exempt from review by the Institutional Review Board of the Chi Mei Medical Center.

### Selection of Patients and Variables

**Figure 1:**
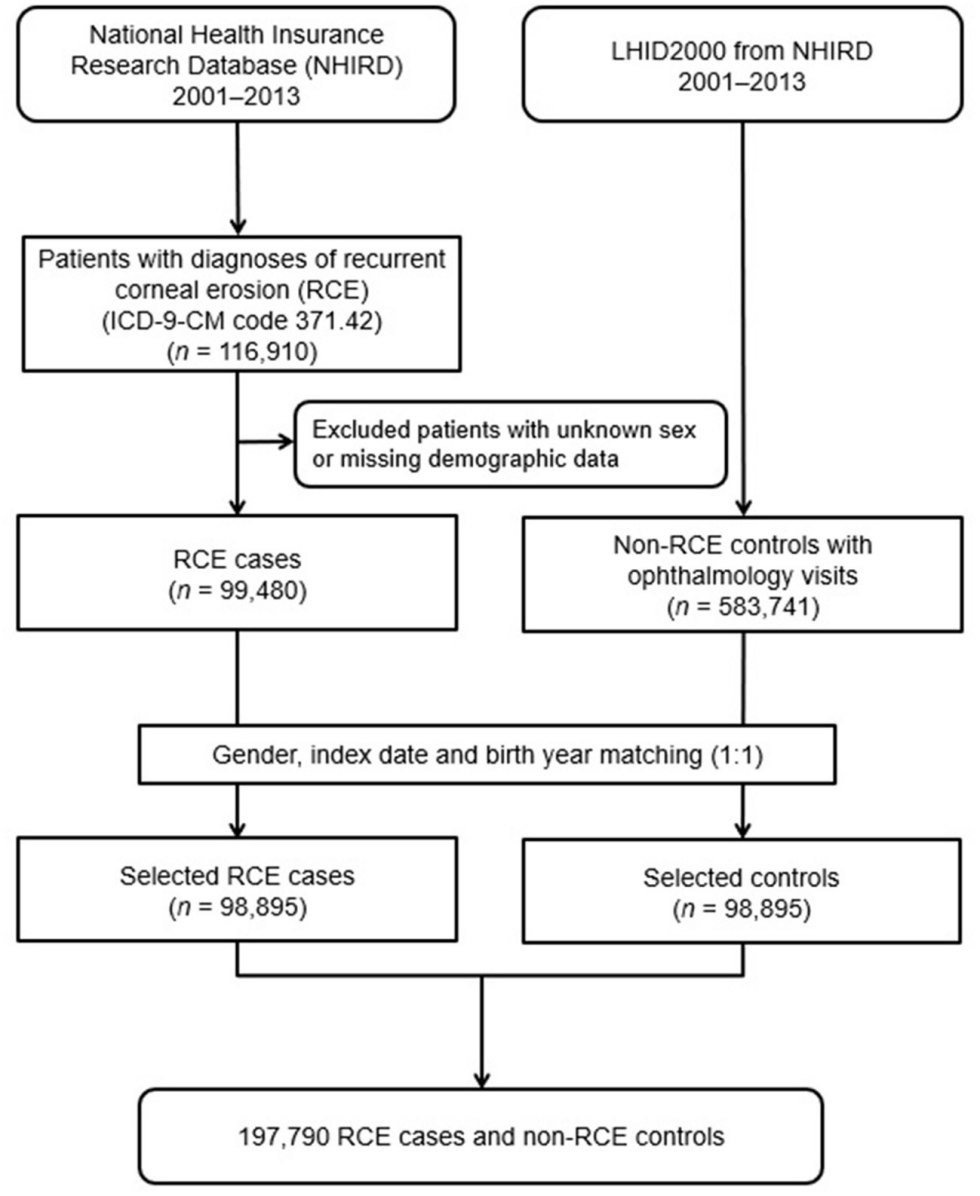
Flowchart demonstrating the enrolment of patients with RCE and the control patients.

A diagnosed RCE group and a matched non-RCE control group were enrolled in this population-based case-controlled study. The patient information from both groups were collected from January 1, 2001, to December 31, 2013. Figure 1 shows a flowchart of our study. Initially, 116,910 patients diagnosed with RCE (ICD-9-CM code 371.42) were included in the study. From these, a total of 99,480 patients were enrolled after we excluded 17,430 patients with missing demographic data on income, region, residential city status, or occupation. Therefore, 98,895 patients diagnosed with RCE taken from the NHIRD were finally included after matching with the controls in the study.

For each patient with RCE, one non-RCE control was randomly chosen from the Longitudinal Health Insurance Database 2000 (LHID2000), which is a subset of the NHIRD that contained the claim data for one million beneficiaries for the year 2000. Initially, we included 583,741 subjects who had reported at least one visit to the ophthalmologist and did not have an RCE diagnosis before the index date from the one million subjects recorded in the LHID2000, after excluding patients with missing sex or demographic data. The control group (*n* = 98,895) was matched with RCE patients by age (±30 days), sex, and index date, defined as the first day of diagnosis with RCE. Each participant was tracked, and their demographic data were recorded from the index date until the end of 2013 or death, whichever was earlier.

The residential cities were classified as metropolis (Taipei City, New Taipei City, Taoyuan City, Taichung City, Tainan City, and Kaohsiung City), satellite (Keelung City, Hsinchu City, Chiayi City), and rural (others). The income levels were identified as <NT$ 30,000, NT$ 30,000–60,000, NT$ 60,000–90,000, NT$ 90,000–120,000, and >NT$ 120,000 based on the fees paid by beneficiaries. The occupational status of NHIRD included beneficiaries from government, school, private enterprise, occupational member, farmer and fishermen, low-income household, and veterans. Thus, we classified them as public servant, farmer, fisherman, and others in our study.

To determine the medical comorbidities for RCE, data regarding comorbid conditions such as DM (ICD-9-CM code 250), hyperlipidaemia (ICD-9-CM code 272), atopy trait [asthma (ICD-9-CM code 493), allergic rhinitis (ICD-9-CM code 477), and atopic dermatitis (ICD-9-CM code 691)], corneal abrasion, ocular allergic conditions [allergic conjunctivitis (ICD-9-CM code 372.14), atopic keratoconjunctivitis (ICD-9-CM code 372.05), and vernal keratoconjunctivitis (ICD-9-CM code 372.13, 370.32)], corneal dystrophy (ICD-9-CM code 371.5), corneal transplantation (order codes 85212B, 85213B, 85215B, 85216B, and 85217B), corneal oedema or disorder due to contact lens (ICD-9-CM code 371.24 and 371.82), and band keratopathy (ICD-9-CM code 371.43) were collected. These comorbidities were identified based on an ICD-9-CM code being recorded within 1 year before the index date and ascertained by one or more ambulatory care claims or admittance as an inpatient.

### Statistical Analysis

All statistical analyses were performed using the Statistical Analysis System 9.4 for Windows (SAS Institute, Inc., Cary, North Carolina, USA). Demographic characteristics such as age group, sex, income, geographic region, city of residence, and occupation were analyzed using McNemar's test, and continuous variables were calculated using the paired *t*-test. The comorbid conditions (DM, hyperlipidaemia, atopy trait, corneal abrasion, ocular allergic condition, corneal dystrophy, corneal transplantation, contact lens, and band keratopathy) were compared between RCE patients and the control group using McNemar's test. Odds Ratios (ORs) obtained by univariate logistic regression analyses and a multivariable logistic regression model (conditional on age, sex, and index date) were constructed to compute the adjusted OR of various comorbidities with a diagnosis of RCE. The independent variables included sociodemographic factors (income, geographic region, residential city status, and occupation) and all medical conditions stated. The level of significance was set at *p* < 0.05.

## Results

### Demographic Data

After ineligible patients were excluded, 98,895 patients with RCE and 98,895 age and sex- matched control group who had used medical care services covered by the NHI between 2001 and 2013 were analyzed. The mean age of both groups were 44.55 (standard deviation = 16.42) (Table 1). Among the 98,895 patients with RCE, the number and percentage of patients in each age category are shown in Table 1. Among the 98,895 patients with RCE, 39,703 (40.15%) were men and 59,492 (59.85%) were women. The incomes of RCE patients were significantly different from those of the control group. The most common approximate income of RCE patients was <30,000 New Taiwan dollars (NT$) (50,052; 50.61%) (*p* < 0.0001). With regards to geographic distribution, the most common region of residence among patients diagnosed with RCE was Northern Taiwan (78,552; 79.43%) and most RCE patients resided in a metropolis city (83,588; 84.52%) with a significant difference from the control group (*p* < 0.0001). With regards to occupation, a significant difference was found between the two groups; over half of the 98,895 RCE patients were public servants, including military, civil, or teaching staff (64,168; 64.88%) (*p* < 0.0001).

**Table 1:** Baseline sociodemographic factors and comorbid conditions of recurrent corneal erosion patients and control subjects after matching by age and gender.

	**Recurrent corneal erosion *N* = 98,895**	**Comparison *N* = 98,895**	***P-*value**
**Sociodemographic factors**	*n* (%)	*n* (%)	
Age (year; Mean ± SD)	44.55 ± 16.42	44.55 ± 16.42	1.0000^a^
Age (year)
<25	11,167 (11.29)	11,167 (11.29)	1.0000^b^
25–34	20,073 (20.30)	20,073 (20.30)	
35–44	19,287 (19.50)	19,287 (19.50)	
45–54	20,992 (21.23)	20,992 (21.23)	
55–64	14,936 (15.10)	14,936 (15.10)	
≥65	12,440 (12.58)	12,440 (12.58)	
Gender
Male	39,703 (40.15)	39,703 (40.15)	1.0000^b^
Female	59,492 (59.85)	59,492 (59.85)	
Income			<0.0001^b^
< NT$ 30,000	50,052 (50.61)	59,533 (60.20)	
NT$ 30,000–60,000	37,569 (37.99)	31,944 (32.30)	
NT$ 60,000–90,000	8,083 (8.17)	5,697 (5.76)	
NT$ 90,000–120,000	1,698 (1.72)	933 (0.94)	
>NT$ 120,000	1,493 (1.51)	788 (0.80)	
Geographical region of Taiwan			<0.0001^b^
Northern	78,552 (79.43)	51,214 (51.79)	
Central	8,849 (8.95)	18,747 (18.96)	
Southern	9,150 (9.25)	26,348 (26.64)	
Eastern	2,344 (2.37)	2,586 (2.61)	
Residential city status			<0.0001^b^
Metropolis	83,585 (84.52)	71,923 (72.73)	
Satellite	4,877 (4.93)	6,876 (6.95)	
Rural	10,433 (10.55)	20,096 (20.32)	
Occupation			<0.0001^b^
Public servant	64,168 (64.88)	57,518 (58.16)	
Farmer	4,711 (4.76)	10,170 (10.28)	
Fisherman	931 (0.94)	1,840 (1.86)	
Other	29,085 (29.41)	29,367 (29.70)	
**Comorbid conditions**
Diabetes mellitus	8,188 (8.28)	5,548 (5.61)	<0.0001^b^
Hyperlipidemia	7,868 (7.96)	4,728 (4.78)	<0.0001^b^
Atopy trait	11,809 (11.94)	8,463 (8.56)	<0.0001^b^
Corneal abrasion	1,549 (1.57)	25 (0.03)	<0.0001^b^
Ocular allergic condition	4,157 (4.20)	153 (0.15)	<0.0001^b^
Corneal dystrophy	54 (0.05)	3 (0.00)	<0.0001^b^
Corneal transplantation	167 (0.17)	0 (0.00)	<0.0001^b^
Contact lens	48 (0.05)	0 (0.00)	<0.0001^b^
Band keratopathy	23 (0.02)	0 (0.00)	<0.0001^b^

a *Paired t-test*;

b *McNemar's test*.

*NT$, New Taiwan dollars; SD, standard deviation*.

There were significant higher prevalences of the possible comorbidities such as DM (8,188; 8.28%), hyperlipidaemia (7,868; 7.96%), atopy trait (11,809; 11.94%), corneal abrasion (1,549; 1.57%), ocular allergic condition (4,157; 4.20%), corneal dystrophy (54; 0.05%), corneal transplantation (167; 0.17%), contact lens (48; 0.05%), and band keratopathy (23; 0.02%), than the control group (*p* < 0.0001) (Table 1).

### Associated Risk Factors

Sociodemographic factors of the RCE patients and the control group were examined using univariate logistic regression analyses and a multiple logistic regression model with adjustments for age, sex, sociodemographic factors, and comorbidities (Table 2). Patients whose income ≥ NT$ 30,000 had increased odds of developing RCE relative to those with an income < NT$ 30,000. A higher income continued to be a significant risk factor for RCE after adjustment for other confounders. Patients who lived in Northern Taiwan (OR = 1.72, 95% confidence interval [CI] = 1.62–1.82, *p* < 0.0001; adjusted OR = 1.23, 95% CI = 1.15–1.32, *p* < 0.0001) or a metropolis city showed a significantly higher prevalence of RCE relative to those who lived in Eastern Taiwan or a rural area, and remained a significant risk factor after a conditional logistic regression analysis was conducted.

**Table 2:** Odds ratios and adjusted odds ratios for various sociodemographic factors and comorbid conditions with recurrent corneal erosion.

	**Odds Ratio^a^ (95% CI)**	***P-*value**	**Adjusted Odds Ratio^b^ (95% CI)**	***P-*value**
**Sociodemographic factors**
Income
< NT$ 30,000	1.00		1.00	
NT$ 30,000–60,000	1.49 (146–1.52)	<0.0001	1.25 (1.22–1.27)	<0.0001
NT$ 60,000–90,000	1.84 (1.77–1.91)	<0.0001	1.48 (1.42–1.55)	<0.0001
NT$ 90,000–120,000	2.38 (2.19–2.58)	<0.0001	1.81 (1.65–1.98)	<0.0001
>NT$ 120,000	2.49 (2.27–2.72)	<0.0001	1.77 (1.61–1.94)	<0.0001
Geographical region of Taiwan
Northern	1.72 (1.62–1.82)	<0.0001	1.23 (1.15–1.32)	<0.0001
Central	0.52 (0.49–0.55)	<0.0001	0.43 (0.40–0.46)	<0.0001
Southern	0.38 (0.36–0.41)	<0.0001	0.30 (0.28–0.32)	<0.0001
Eastern	1.00		1.00	
Residential city status
Metropolis	1.00		1.00	
Satellite	0.61 (0.59–0.63)	<0.0001	0.56 (0.53–0.58)	<0.0001
Rural	0.44 (0.43–0.45)	<0.0001	0.91 (0.88–0.94)	<0.0001
Occupation
Public servant	1.19 (1.17–1.22)	<0.0001	0.95 (0.92–0.97)	<0.0001
Farmer	0.41 (0.40–0.43)	<0.0001	0.74 (0.70–0.77)	<0.0001
Fisherman	0.53 (0.49–0.57)	<0.0001	0.90 (0.82–0.99)	0.0238
Other	1.00		1.00	
**Comorbid conditions**
Diabetes mellitus	1.58 (1.52–1.64)	<0.0001	1.54 (1.39–1.51)	<0.0001
Hyperlipidemia	1.79 (1.72–1.86)	<0.0001	1.48 (1.41–1.54)	<0.0001
Atopy trait	1.46 (1.42–1.50)	<0.0001	1.37 (1.33–1.42)	<0.0001
Corneal abrasion	64.50 (43.10–96.53)	<0.0001	63.56 (42.06–96.06)	<0.0001
Ocular allergic condition	28.05 (23.81–33.05)	<0.0001	24.27 (20.51–28.72)	<0.0001
Corneal dystrophy	18.00 (5.63–57.57)	<0.0001	17.10 (5.14–59.93)	<0.0001

a *Odds ratio was obtained from a univariate logistic regression analysis*;

b *Adjusted odds ratio was calculated from a multivariable logistic regression model that was conditioned on age-group, sex, and the year of index date*.

*NT$, New Taiwan dollars; CI, confidence interval*.

Several possible comorbidities were also examined using univariate and multiple logistic regression analyses (Table 2). Patients with ocular conditions such as corneal abrasion, ocular allergic condition, and corneal dystrophy had significantly higher ORs of receiving a diagnosis of RCE (OR = 64.50, 95% CI = 43.10–96.53, *p* < 0.0001; OR = 28.05, 95% CI = 23.81–33.05, *p* < 0.0001; OR = 18.00, 95% CI = 5.63–57.57, *p* < 0.0001, respectively) even after conditional logistic regression was conducted (adjusted OR = 63.56, 95% CI = 42.06–96.06, *p* < 0.0001; adjusted OR = 24.27, 95% CI = 20.51–28.72, *p* < 0.0001; adjusted OR = 17.10, 95% CI = 5.14–59.93, *p* < 0.0001, respectively). Patients with DM, hyperlipidaemia, and atopy trait (asthma, allergic rhinitis, atopic dermatitis) had significantly higher odds of a RCE diagnosis before and after adjustment for other confounders (OR = 1.58, 95% CI = 1.52–1.64, *p* < 0.0001, adjusted OR = 1.54, 95% CI=1.39–1.51, *p* < 0.0001; OR = 1.79, 95% CI = 1.72–1.86, *p* < 0.0001, adjusted OR=1.48, 95% CI = 1.41–1.54, *p* < 0.0001; OR = 1.46, 95% CI = 1.42–1.50, *p* < 0.0001, adjusted OR = 1.37, 95% CI = 1.33–1.42, *p* < 0.0001, respectively).

## Discussion

To the best of our knowledge, this study appears to be the largest nationwide, Taiwan population-based, case-controlled study that evaluates the association between sociodemographic factors, common comorbid conditions, and RCE. These analyses identified several key findings. First, more than half of the RCE patients in Taiwan were women with a female to male ratio (1.49:1). Second, the odds of developing RCE varied with sociodemographic factors; an income ≥ NT$ 30,000 and residence in either Northern Taiwan or a metropolis city had higher odds of developing RCE. Third, some comorbid conditions significantly increased the odds of developing RCE including of DM, hyperlipidaemia, atopy trait, corneal abrasion, ocular allergic condition, and corneal dystrophy. We were unable to study whether age and sex affected the risk of developing RCE. The RCE patients demonstrate a female preponderance consistent with previous reports (9–11). Among the sociodemographic factors, we found patients living in Northern Taiwan and metropolis areas show statistically significant associations to RCE development. Higher rates of RCE diagnosis in Northern Taiwan and metropolis areas may be due to the accessibility to ophthalmologists, and enhanced ease of access to corneal specialists for diagnosis and management of RCE, compared with other regions of Taiwan and other residential city statuses. In addition, we also observed that Central and Southern Taiwan had significantly lower risk of REC when compared with Eastern Taiwan. We contributed the association between patients living in Eastern Taiwan and RCE compared with the Central and Southern Taiwan to the different environment in the Eastern Taiwan. Eastern Taiwan particularly has a stretch long coastline, and the humid and windy coastal climate may lead to dryness of the ocular surface. Frequent eye rubbing due to discomfort in the eyes may lead to an ocular surface injury in people living in Eastern Taiwan, which is a possible risk factor for the development of RCE compared with Central and Southern Taiwan. We also found a significant association between RCE and individuals with an income ≥NT$ 30,000 although the NHI program improves the accessibility of medical care service for all population in Taiwan, and that increases the medical utilization in low-income population. We attempt to explain the association between RCE and higher income based on two reasons. First, people with higher income may have more knowledge and awareness about corneal disorder than those with lower income (12). Second, people with higher income would immediately and actively seek help if corneal trauma occurred (12). The awareness and actions may lead to more ophthalmology visiting and more RCE diagnosis in people with higher income than those with lower income. Compared with most workers, people with occupations (including public service, farming, or fishing) have less odds to have RCE, probably implying that RCE occurs due to multiple triggers, including genes, comorbidities, environmental factors, and behavioral factors, rather than only preference for outdoor or indoor job style. We reported that patients with corneal abrasion had a significantly higher risk of developing RCE than the control group (adjusted OR = 63.56, 95% CI = 42.06–96.06, *p* < 0.0001), in accordance with previous studies (1–3, 5). Nanba et al. (1) conducted a retrospective study including 21 eyes of 21 patients with RCE and found that more than 47% had ocular trauma history, implying that ocular trauma is an important risk factor for RCE. The most common cause of RCE related to corneal abrasion is fingernail scratches followed by paper cuts, corneal foreign bodies, or tree branch injuries (1, 3). A compromised ocular surface with epithelial superficial squamous cell damage could also result to RCE.

Patients with ocular allergic conditions had a remarkably higher OR for RCE development (adjusted OR = 24.27, 95% CI = 20.51–28.72, *p* < 0.0001). This finding is consistent with a previous, retrospective, nationwide, matched cohort study that included 184,166 patients with atopic keratoconjunctivitis who were 1.36 times (95% CI = 1.19–1.54) more likely to develop RCE than the control group (8). The high concentration of eosinophil granular substances in patients with ocular allergic conditions may be a possible explanation (13–15) for its association with RCE (16, 17). Another explanation for the association includes frequent eye rubbing, which is a common physiological response to fatigue, itching, and discomfort in patients with ocular allergic conditions (18, 19). Eye rubbing causes ocular surface injury, elevated inflammatory cytokines (5, 6), and increased expression of extracellular matrix proteins (20–22), which play a role in the pathophysiology of RCE.

In addition, patients with corneal dystrophy had a significantly higher OR for RCE development (adjusted OR = 17.10, 95% CI = 5.14–59.93, *p* < 0.0001). The relationship between RCE and corneal dystrophy is well-established (23, 24). Laibson reported that desquamating cells were entrapped beneath redundant layers of basement membrane in patients with corneal dystrophy, leading to poor adherence between the corneal epithelium and underlying adhesion network (24). In addition, Rosenberg et al. showed the common manifestations of *in vivo* confocal microscopy in patients with RCE and epithelial basement membrane dystrophy, such as sub- basal microfolds and streaks, basal epithelial cells deposits, and damaged subbasal nerves (23).

DM is an independent risk factor for RCE after adjusting for other confounders. The finding is consistent with several previous studies (1, 4, 25). Jan et al. included 239,854 patients with DM and showed that DM was an independent risk factor of RCE (adjusted hazard ratio 1.35 [95% CI = 1.24–1.48]) in the total sample cohort. The risk for RCE due to DM may be explained by the accumulation of advanced glycation end products due to hyperglycaemia (26), epithelial basement membrane abnormalities (27), and decreased sub-basal nerve density in the cornea (28). These changes in corneal morphology and pathophysiology among patients with DM were also found in patients with RCE, indicating the important role of the systemic disease in RCE development (2, 4, 29).

Hyperlipidaemia was another significant risk factor of RCE, in accordance with a previous report (25). We attempt to explain that the association between hyperlipidaemia and RCE through the influence of hyperlipidaemia to corneal nerve fiber regeneration (30). In addition, several corneal lipid deposition such as lipid keratopathy, corneal arcus, and crystalline stromal dystrophy resulted from hyperlipidaemia (31, 32). Therefore, we suggest that eye rubbing due to corneal lipid deposition explains the association of hyperlipidaemia with RCE.

Our study had several strengths. The current study focuses on RCE patients, with 98,895 cases identified in the NHIRD database. Since the data obtained was based on a nationwide and population-based dataset, the selection bias regarding referral centers was reduced. In addition, recall bias was reduced as the claims data of the NHIRD were electronically recorded and were not reliant on self-reports. Furthermore, our study was case-controlled and incorporated 13 years of longitudinal data on various sociodemographic factors, and comorbid conditions in RCE patients and the control group. Our results are also reliable, because there was appropriate adjustment for potential confounding factors such as sociodemographic factors and comorbid conditions, including DM, hyperlipidaemia, atopy trait, corneal abrasion, ocular allergic condition, corneal dystrophy, corneal transplantation, contact lens, and band keratopathy.

This study had several limitations. The diagnosis of RCE and other comorbid disorders may be misclassified because the diagnosis was based on ICD-9-CM codes. In addition, the presence of RCE in the patients or the absence of RCE in the control group were based on the claims data without access to clinical records, which may lead to uncertainty. Second, there was no information to confirm that the control group had not been diagnosed with RCE before January 1996, since the medical records could only be traced back to 1996. Finally, we could not determine whether ocular blunt trauma, corneal dystrophy, or band keratopathy were significant risk factors of RCE. These conditions were not present in the control group; hence, it could compromise our results. In addition, fewer numbers of corneal abrasion, ocular allergic condition, and corneal dystrophy made the non-convergence odds ratios in our results. Therefore, the results should not be an over-interpretation. We will keep it in mind in our future research.

In summary, sociodemographic factors including an income ≥ NT$ 30,000 and residence in either Northern Taiwan or a metropolis city were associated with an increased risk of RCE. After controlling for sociodemographic factors and comorbidities, patients with corneal abrasion, ocular allergic conditions, and corneal dystrophy as well as DM, hyperlipidaemia, and atopy trait had significantly higher risks of developing RCE than the control group. This association may help clinicians understand the pathophysiology of RCE.

## Data Availability Statement

The original contributions presented in the study are included in the article/supplementary material, further inquiries can be directed to the corresponding author.

## Author Contributions

R-LJ, S-HT, and Y-SC conducted the study. R-LJ, C-HH, and Y-SC analyzed the results. C-HH and J-JW provided materials. R-LJ and Y-SC wrote the article. All authors reviewed the manuscript and conceived the study.

## Conflict of Interest

The authors declare that the research was conducted in the absence of any commercial or financial relationships that could be construed as a potential conflict of interest.

## Publisher's Note

All claims expressed in this article are solely those of the authors and do not necessarily represent those of their affiliated organizations, or those of the publisher, the editors and the reviewers. Any product that may be evaluated in this article, or claim that may be made by its manufacturer, is not guaranteed or endorsed by the publisher.

## Abbreviations

CI: confidence interval
DM: diabetes mellitus
ICD-9-CM: International Classification of Diseases, Ninth Revision, Clinical Modification
IRB: Institutional Review Board
LHID2000: Longitudinal Health Insurance Database 2000
NHIRD: National Health Insurance Research Database
NHRI: National Health Research Institute
NT$: New Taiwan dollars
OR: odds ratio
RCE: recurrent corneal erosion
SD: standard deviation.
